# Early molecular markers of ventilator-associated pneumonia in bronchoalveolar lavage in preterm infants

**DOI:** 10.1038/s41390-022-02271-w

**Published:** 2022-09-07

**Authors:** Alejandro Pinilla-Gonzalez, Inmaculada Lara-Cantón, Laura Torrejón-Rodríguez, Anna Parra-Llorca, Marta Aguar, Julia Kuligowski, José David Piñeiro-Ramos, Ángel Sánchez-Illana, Ana Gimeno Navarro, Máximo Vento, María Cernada

**Affiliations:** 1 Division of Neonatology, University and Polytechnic Hospital La Fe (HULAFE), Valencia, Spain; 2 Neonatal Research Group, Health Research Institute La Fe (IISLAFE), Valencia, Spain; 3 National Coordinator of the Spanish Maternal and Infant Health and Development Network, Health Research Institute Carlos III, Spanish Ministry of Economy and Competitiveness (RD12/0026), Valencia, Spain; 4 Present Address: Analytical Chemistry Department, University of Valencia, Burjassot, Spain

## Abstract

**Introduction:**

Ventilator-associated pneumonia (VAP) constitutes a serious nosocomial infection. Our aim was to evaluate the reliability of cytokines and oxidative stress/inflammation biomarkers in bronchoalveolar lavage fluid (BALF) and tracheal aspirates (TA) as early biomarkers of VAP in preterm infants.

**Methods:**

Two cohorts were enrolled, one to select candidates and the other for validation. In both, we included preterms with suspected VAP, according to BALF culture, they were classified into confirmed VAP and no VAP. Concentration of 16 cytokines and 8 oxidative stress/inflammation biomarkers in BALF and TA was determined in all patients.

**Results:**

In the first batch, IL-17A and TNF-α in BALF, and in the second one IL-10, IL-6, and TNF-α in BALF were significantly higher in VAP patients. BALF TNF-α AUC in both cohorts was 0.86 (sensitivity 0.83, specificity 0.88). No cytokine was shown to be predictive of VAP in TA. A statistically significant increase in the VAP group was found for glutathione sulfonamide (GSA) in BALF and TA.

**Conclusions:**

TNF-α in BALF and GSA in BALF and TA were associated with VAP in preterm newborns; thus, they could be used as early biomarkers of VAP. Further studies with an increased number of patients are needed to confirm these results.

**Impact:**

- We found that TNF-α BALF and GSA in both BALF and TA are capable of discriminating preterm infants with VAP from those with pulmonary pathology without infection.
- This is the first study in preterm infants aiming to evaluate the reliability of cytokines and oxidative stress/inflammation biomarkers in BALF and TA as early diagnostic markers of VAP. We have validated these results in two independent cohorts of patients. Previously studies have focused on full-term neonates and toddlers and determined biomarkers mostly in TA, but none was exclusively conducted in preterm infants.

## Introduction

Improvement of mechanical ventilation (MV) has substantially increased the survival of very preterm infants (<32 weeks gestation). However, MV is inevitably associated with significant mortality and morbidity such as bronchopulmonary dysplasia (BPD) and ventilator-associated pneumonia (VAP).^1^ VAP is defined as a nosocomial lung infection diagnosed in patients undergoing MV for at least 48 h^2^ and is considered the second most frequent cause of nosocomial infection in neonatal and pediatric intensive care patients.

In developed countries, the rate of neonatal VAP has been reported to vary between 2.1 and 10.9 episodes/1000 ventilator days.^3^ Moreover, in developing countries the incidence can reach up to 37.2 episodes/1000 ventilator days.^4^ Of note, the duration of MV has been identified as the main independent risk factor for VAP.^3^ The major concern for neonatologists, reflected by the wide differences in VAP incidence, resides in the lack of reliable diagnostic tools or biomarkers. The criteria established by the Center for Disease Control (CDC) for the diagnosis of VAP are referred non-specifically to infants <1 year and require a combination of clinical and radiological signs in addition to the isolation of a microorganism in bronchoalveolar lavage fluid (BALF).^5^ However, the most susceptible infants to develop VAP are very preterm infants undergoing MV for long periods of time in the context of BPD with radiographic involvement at baseline and episodes of clinical worsening. Thus, clinical/radiological diagnosis of VAP in preterm newborns with BPD is complex and requires strict microbiological criteria to avoid overdiagnosis.^6^ In addition, pre-treatment with antibiotics when VAP is suspected frequently yields false negatives.^7^ Furthermore, microbiological results are generally delayed more than 48 h, rendering them helpless to early start antibiotic therapy.^8^

The main drawback of VAP diagnosis is the difficulty in getting non-contaminated samples from the infant’s lower airways and the lack of accurate biomarkers of VAP in peripheral blood or BALF. We have previously described the use of BALF lavage collected using a brush-protected catheter as a reliable tool for diagnosing VAP.^3^ However, this technique requires highly trained personnel, especially in very preterm infants, to avoid complications during the procedure. In adult patients, the use of certain cytokines (TNF-α, IL-1α, IL-8, and IL-6) in BALF has been suggested for the diagnosis of VAP. Moreover, the monitoring of IL-8 and TNF-α values has been proposed as an evaluation of the response to treatment.^9,10^ On the other hand, high values of glutathione sulfonamide (GSA) in tracheal aspirates have been associated with a lung infection in neonates.^11^ The aim of the present study was to test the predictive capacity for diagnosing VAP of an ample array of cytokines and biomarkers of oxidative stress and inflammation determined in BALF and TA in preterm newborns. In addition, the correlation between cytokine levels in BALF and TA was assessed since access to the latter is technically easier.

## Methods

### Setting and patients

This was a prospective observational double cohort study carried out in the NICU of the University and Polytechnic Hospital La Fe (HULAFE; Valencia; Spain). The first cohort was recruited from January to December 2013 (*n* = 13), and the second one was from May 2015 to October 2016 (*n* = 15). Samples from the first cohort were processed and analyzed to identify potential biomarkers and samples from the second cohort were collected to validate the results.

Eligible patients were preterm newborns <34 weeks of gestational age with a pulmonary disease who were on MV for more than 48 h and were suspicious of having VAP. The Ethics Committee for Clinical Research of the HULAFE approved the study and all parents signed the informed consent. Exclusion criteria were lack of informed consent, clinical instability to obtain the sample, or airway malformations.

### Definitions and collection of sample

Patients were ventilated in synchronized intermittent positive pressure or pressure support ventilation modes with volume-guarantee using Babylog^®^ or VN500 Dräger^®^ ventilators (Dräger AG, Lübeck, Germany). One set of disposable ventilator circuits was used for each patient, and secretions were suctioned by open method using sterile suction catheters. Definition and diagnostic criteria for VAP were based on CDC guidelines^2^ and are shown in Table 1.

**Table 1:** Diagnostic criteria for ventilation-associated pneumonia in mechanically ventilated neonates according to the Centers for Disease Control and Prevention/National Nosocomial Infection Surveillance criteria for patients younger than 12 months of age.

Type	Criteria
	>48 h of mechanical ventilation
Radiologic	Persistent radiological infiltrates or consolidation in two sequential radiographs after initiation of mechanical ventilation
Clinical	Worsening of the respiratory conditions characterized by increased
	• need for supplemental oxygen
	• respirator settings to achieve targeted respiratory values
	• amount of respiratory secretions
	• incidence of desaturation events
	Presence of tachypnea, apnea, and/or retractions
Microbiological	Isolation of any microorganism with more than 1000 CFU/mL in bronchoalveolar lavage fluid

Only patients presenting clinical and radiological criteria underwent BAL before starting antibiotic treatment. A total of 1 mL of BALF was extracted with a brush-protected catheter (Combicath^®^, Plastimed, Saint Leu la Forêt, France) following a strict sterile procedure previously described: usual infection control precautions (sterile gloves, mask and cap, single-use protective clothing), insert the Combicath^®^ in the closed position, extend the inner catheter to expel the plug, push the inner catheter through until it reaches the guard and inject 1–2 mL of saline solution, and re-aspirate the mini-lavage saline solution for analysis.^3^ An aliquot of 0.5 mL was planted on blood agar media and chocolate agar media for microbiologic culture and the remaining 0.5 mL were stored at −80 °C until processing. BALF quantitative culture was considered positive for VAP when more than 1000 CFU/mL were isolated. Colony characteristics were observed, and identification was done according to routine microbiologic procedures.

**Fig. 1:**
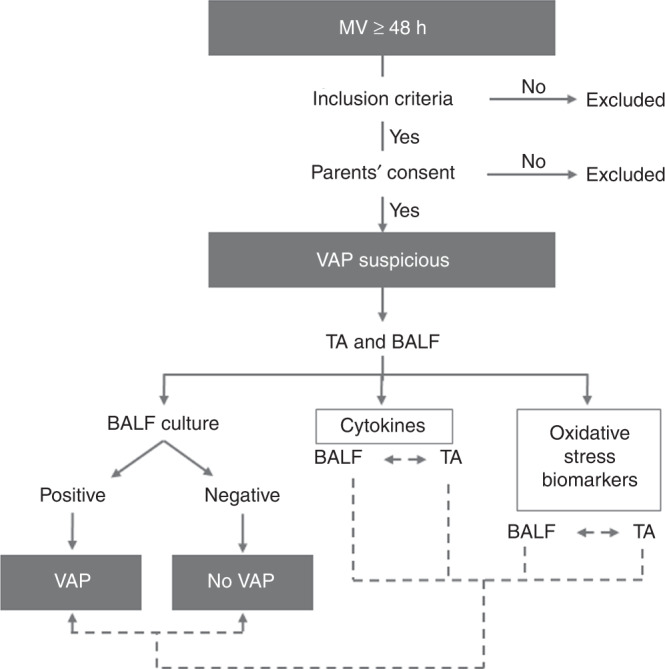
Workflow chart. Dynamic of recruitment and sample collection and analysis. MV mechanical ventilation, VAP ventilator-associated pneumonia, TA tracheal aspirate, BALF bronchoalveolar lavage fluid.

A TA sample was obtained by suctioning through an endotracheal tube after BALF technique had been performed and stored at –80 °C until processing. Figure 1 represents the flow diagram of recruitment and sample collection and analysis.

When BALF culture was negative, the antibiotic treatment was immediately stopped, and when positive, tests were scheduled to characterize the causative microorganism to adjust antibiotic treatment.

### Measurement of cytokines in BALF and TA

The samples were analyzed in two different batches employing commercial bead-based immunoassays kits. Two different kits were used to check the reproducibility of the obtained results. For the first batch (*n* = 13, cohort I), Human RTU FlowCytomix from eBiosience Inc (San Diego, CA) was employed, while for the second batch (*n* = 15, cohort II) Cytometric Bead Array kit from BD Biosciences (San Jose, CA) was used. With both kits, concentrations of 16 human cytokines (pg/mL) including *E-Selectin* (*CD62E*), granulocyte-colony stimulating factor (*G-CSF*), intercellular adhesion molecule 1 (*ICAM-1*), interferons α (*IFN-* α*)* and γ (*IFN-γ*), monocyte chemoattractant protein-1 (*MCP-1*), macrophage inflammatory proteins α (*MIP-*α), tumor necrosis factor-alpha (*TNF-*α), and the interleukins *IL-1* α, *IL-4*, *IL-6*, *IL-8*, *IL-10*, *IL-12p70*, *IL-13*, and *IL-17A* were measured following the manufacturers’ instructions in 25 µL of BALF and 25 µL of TA. Data acquisition was performed with a flow cytometry BD FACSVerse^TM^ (San Jose, CA). Measured data were analyzed employing FlowCytomix Pro 2.1^TM^ (Bender MedSystems GmbH, Vienna, Austria). The cytokine levels were normalized with the total protein content measured by Bradford Method (Bio-Rad, Hercules, CA) following the manufacturer’s instructions.

### Determination of molecular biomarkers employing ultra-performance liquid chromatography coupled to tandem mass spectrometry

The excess volume from BALF and TA was processed by freeze drying followed by deproteinization and analyzed by ultra-performance liquid chromatography coupled to tandem mass spectrometry for the determination of a panel of oxidative stress and inflammation biomarkers, including cystine, glutathione (GSH), glutathione disulfide (GSSG), methionine (Met), S-adenosylmethionine (SAM), 3-Cl-tyrosine (3Cl-Tyr), 3-NO_2_-tyrosine (3NO_2_-Tyr), and GSA. For more details, the reader is referred to the Supplementary material.

### Statistical analysis

Data analysis was performed using Matlab 2017b from Mathworks Inc. (Nattick, MA) and MetaboAnalyst 4.0. The area under the curve (AUC) of the receiver operator characteristic (ROC) curve was calculated from autoscaled data to evaluate the discriminant capacity of biomarker candidates, as well as sensitivity, specificity, predictive values, likelihood ratios, and Youden Index. Qualitative variables were expressed as a percentage and quantitative variables as a mean and standard deviation or median according to the distribution of the data. The Kolmogorov–Smirnov test was performed to evaluate the normal distribution of variables. Statistical comparisons between the quantitative variables of the study groups were made using the *χ*^2^, Student’s *t*-test, or Wilcoxon rank-sum test according to the type of variables and the distribution of the data. Spearman’s rank correlation coefficient was used to assess the correlation between levels of cytokines and oxidative stress biomarkers in BALF and TA. All tests of significance were two tailed. A *p* value ≤0.05 was considered significant.

## Results

Twenty-eight preterm infants with pulmonary disease and suspected VAP were enrolled. The first cohort included 13 neonates and the second cohort 15. Out of these, 5 (38%) and 8 (53%), respectively, were diagnosed with VAP. Demographic data followed a normal distribution. No significant demographic and clinical differences were observed between cases and controls (Table 2).

**Table 2:** Clinical characteristics and demographic data in patients with and without ventilator-associated pneumonia (VAP).

Variables	No VAP (*N* = 15)	VAP (*N* = 13)	*P* value
Gestational age (weeks)	26 (24–29)	28 (25–34)	0.09*
Gender, *n* (%)			0.14**
Male	5 (33)	8 (62)	
Female	10 (67)	5 (38)	
Birth weight (g)	760 (665–1260)	1175 (835–2075)	0.12*
APGAR 1 min	4 (3–6)	7 (4–9)	0.06*
APGAR 5 min	8 (7–9)	8 (7–10)	0.3*
Age (days)	13 (8–28)	12 (6–34)	0.8*
Days of MV	10 (5–25)	6 (4–13)	0.2*
Number of reintubations	0 (0–2)	1 (0–1)	0.6*
Central line, *n* (%)	13 (87)	12 (92)	0.6***
Enteral feeds, *n* (%)	10 (67)	10 (77)	0.7***

The continuous variables are expressed as median and interquartile ranges and the discrete variables are expressed as counts and percentages.

*MV* mechanical ventilation.

Statistical analysis: *Student’s *t*-test; **χ^2^ test; ***Fisher’s exact test.

A single pathogen was isolated from BALF in each patient diagnosed with VAP, except for one case with two bacterial strains. Isolated microorganisms were as follows: *Staphylococcus aureus*: n = 3 (23%); *Enterobacter spp*: n = 3 (23%); *Escherichia coli*: n = 2 (15%); *Serratia marcescens*: n = 2 (15%); *Stenotrophomonas maltophilia*: n = 1 (7%); *Pseudomonas aeruginosa*: n = 1 (7%); *Candida albicans*: n = 1 (7%) and *Klebsiella spp* n = 1 (7%), with a median number of colonies per mL of 40000 (5000–100000).

**Fig. 2:**
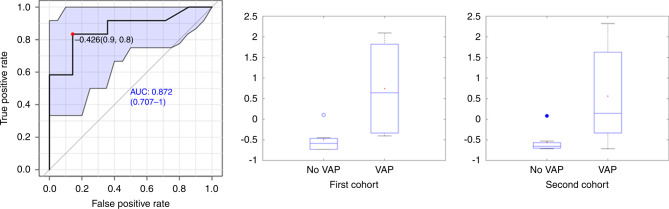
TNF-α in both cohorts. ROC curve TNF-α in BALF (left) and boxplots of VAP vs No VAP samples in the first cohort (middle) and the second cohort (right) (autoscaled data).

A total of 16 cytokines were determined in BALF and TA (Table 3). Cytokine values did not follow a normal distribution. TNFα and IL-17A showed statistically significant higher values in the VAP group in BALF in the first cohort. In the second cohort, the biomarkers with statistically significant higher values in BALF in VAP patients were TNFα, IL-6, and IL-10. Therefore, only TNF-α in BALF showed statistically significant higher values in the VAP group in both cohorts (Table 3). No statistically significant differences were observed for either the remaining cytokines in BALF or any cytokine in TA samples (Table 3). An ROC curve for TNF-α in BALF was calculated with an AUC of 0.87 (Fig. 2) that corresponds to sensitivity value of 0.83, specificity 0.86, positive predictive value 0.83, and negative predictive value 0.86.

**Table 3:** Median (P10–P90) of cytokine concentrations (normalized with the total protein content) in bronchoalveolar lavage fluid (BALF) and tracheal aspirate (TA) in patients with and without ventilator-associated pneumonia (VAP).

	BALF						TA					
	Cohort 1			Cohort 2			Cohort 1			Cohort 2		
Compound	VAP (*N* = 4)	No VAP (*N* = 6)	*P*	VAP (*N* = 8)	No VAP (*N* = 8)	*P*	VAP (*N* = 5)	No VAP (*N* = 6)	*P*	VAP (*N* = 3)	No VAP (*N* = 4)	*P*
IL-12 pg/µg protein	n.d. (n.d. –0.004)	n.d. (n.d. –n.d.)	0.8	0.005 (0.0012–0.04)	0.003 (n.d. −0.009)	0.3	n.d. (n.d. −0.004)	0.007 (n.d. −0.14)	0.2	0.005 (0.004–0.017)	0.006 (0.0008–0.02)	0.9
INF-γ pg/µg protein	1.1 (0.8–1.4)	0.6 (0.2–3)	0.2	0.003 (0.0010–0.02)	0.0009 (n.d. −0.003)	0.10	0.5 (n.d. −1.4)	0.6 (0.14–3)	0.8	0.003 (0.002–0.010)	0.003 (0.0013–0.006)	0.6
G-CSF pg/µg protein	1.0 (0.5–2)	0.4 (n.d. −2.2)	0.3	0.7 (0.03–1.1)	0.10 (n.d. −0.4)	0.06	0.3 (0.10–1.5)	0.6 (0.2–2)	0.7	2 (0.7–3)	0.2 (0.07–0.5)	0.11
MCP-1 pg/µg protein	15 (8–39)	13 (11–17)	0.9	10 (0.3–86)	5 (n.d. −29)	0.3	11 (4–38)	13 (6–24)	1.0	20 (7–28)	4 (0.5–27)	0.6
IL-10 pg/µg protein	0.03 (0.009–0.05)	n.d. (n.d. −0.05)	0.3	0.009 (0.004–0.02)	0.0012 (n.d.–0.007)	0.02	n.d. (n.d.–0.16)	0.03 (n.d. –0.4)	0.4	0.09 (0.04–0.11)	0.0013 (0.0002–0.002)	0.06
MIP-1α pg/µg protein	3 (2–7)	0.7 (0.2–3)	0.07	0.2 (0.11–1.0)	0.08 (0.005–0.8)	0.10	2 (1.0–4)	2 (0.3–3)	0.3	2 (1.4–4)	0.07 (0.02–0.5)	0.06
IL-8 pg/µg protein	14 (8–59)	7 (1.1–27)	0.3	6 (2–20)	13 (0.02–26)	1.0	21 (2–37)	4 (2–21)	0.4	7 (3–7)	6 (1.3–9)	0.9
ICAM-1 ng/µg protein	2 (1.3–4)	2 (1.2–21)	1.0	425 (n.d.–21,865)	27 (n.d. –1,748,731)	0.7	2 (1.1–4)	3 (1.2–14)	0.5	n.d. (n.d.–n.d.)	32 (0.010–349)	0.2
IL-6 pg/µg protein	1.4 (0.6–1.6)	n.d. (n.d. –0.4)	0.14	3 (0.4–8)	0.3 (n.d. –3)	0.05	0.5 (0.2–2)	0.8 (0.14–2)	0.8	22 (9–50)	1.3 (0.08–3)	0.06
IL-1α pg/µg protein	0.3 (0.2–0.3)	0.06 (n.d. –0.4)	0.11	0.2 (0.02–2)	0.02 (n.d. –0.2)	0.08	0.2 (0.12–0.5)	0.12 (0.015–0.4)	0.5	2 (2–5)	0.02 (0.012–0.11)	0.06
INF-α pg/µg protein	0.9 (0.5–1.3)	0.9 (0.4–2)	0.8	0.007 (0.0011–0.6)	0.002 (n.d.–0.006)	0.11	0.4 (0.03–2)	1.0 (0.2–2)	0.7	0.005 (0.005–0.02)	0.002 (0.0003–0.004)	0.06
IL-13 pg/µg protein	0.5 (0.3–0.7)	0.4 (0.08–0.8)	0.5	0.002 (0.0008–0.02)	0.002 (0.0008–0.003)	0.5	0.2 (0.014–0.9)	0.3 (0.09–2)	0.7	0.003 (0.002–0.008)	0.0011 (0.0003–0.002)	0.06
IL-4 pg/µg protein	0.2(0.12–0.3)	0.2 (0.04–0.4)	0.8	0.0013 (0.0004–0.004)	0.0003 (n.d.–0.002)	0.2	0.09 (0.005–0.4)	0.3 (0.05–1.1)	0.2	0.002 (0.0012–0.006)	0.0006 (n.d.–0.0015)	0.2
IL-17 A pg/µg protein	0.5 (0.3–0.7)	0.2 (n.d. –0.3)	0.04	0.007 (0.003–0.016)	0.005 (0.0012–0.02)	0.7	0.3 (0.011–0.9)	0.2 (0.07–1.1)	0.8	0.02 (0.011–0.02)	0.003 (0.0007–0.004)	0.06
TNF-α pg/µg protein	0.7 (0.2–1.3)	0.07 (n.d. –0.3)	0.04	0.06 (0.006–0.2)	0.004 (n.d. –0.03)	0.02	0.4 (0.07–0.5)	0.3 (0.11–0.6)	1.0	0.2 (0.2–5)	0.004 (0.0011–0.008)	0.06
E-SELECTIN ng/µg protein	0.05 (0.03–0.08)	0.05 (0.03–0.12)	1.0	0.04 (0.02–0.11)	0.04 (n.d. –0.09)	0.7	0.07 (0.02–0.2)	0.04 (0.012–0.10)	0.5	0.05 (0.04–0.11)	0.03 (0.007–0.05)	0.2

*n.d.* not detected, *LAP* latency-associated peptide, *IFN-γ* interferon-gamma, *MIP-1β* macrophage inflammatory protein-beta, *G-CSF* granulocyte-colony stimulating factor, *MCP-1* monocyte chemoattractant protein-1, *MIP-1α* macrophage inflammatory protein-alpha, *ICAM-1* intercellular adhesion molecule 1, *IFN-α* interferon-alpha, *IP-10* IFN-γ inducible protein 10, *TNF-α* tumor necrosis factor-alpha.

*P* values correspond to Wilcoxon rank-sum test.

Out of the 16 analyzed cytokines, in the first cohort, only ICAM showed a significant positive correlation between values in BALF and TA. In the second cohort, IL-6, IL-1α, and TNF-α showed a significant positive correlation. No statistically significant correlations were found between values in BALF and TA for the remaining cytokines (Table 4).

**Table 4:** Correlations between cytokine levels in bronchoalveolar lavage fluid (BALF) and tracheal aspirate (TA).

	Cohort 1		Cohort 2	
Cytokines (BALF vs TA)	Spearman’s coefficient	*P* value	Spearman’s coefficient	*P* value
IL-12	−0.4	0.4	0.6	0.14
INF-γ	−0.02	1.0	0.2	0.7
G-CSF	−0.3	0.5	0.2	0.7
MCP-1	−0.05	0.9	0.5	0.2
IL-10	−0.04	0.9	0.8	0.10
MIP-1α	−0.05	0.9	0.6	0.2
IL-8	0.09	0.8	0.3	0.5
ICAM-1	0.7	0.04	0.4	0.4
IL-6	0.5	0.2	0.3	0.002
IL-1α	0.3	0.5	0.8	0.03
INF-α	−0.07	0.9	0.5	0.2
IL-13	0.3	0.5	0.3	0.5
IL-4	0.5	0.2	0.6	0.09
IL-17 A	−0.2	0.7	0.2	0.6
TNF-α	−0.07	0.9	1.0	<0.001
E-selectin	−0.5	0.2	0.5	0.2

*LAP* latency-associated peptide, *IFN-γ* interferon-gamma, *MIP-1β* macrophage inflammatory protein-beta, *G-CSF* granulocyte-colony stimulating factor, *MCP-1* monocyte chemoattractant protein-1, *MIP-1α* macrophage inflammatory protein-alpha, *ICAM-1* intercellular adhesion molecule 1, *IFN-α* interferon-alpha, *IP-10* IFN-γ inducible protein 10, *TNF-α* tumor necrosis factor-alpha.

*P* values correspond to Spearman’s rank correlation coefficient test.

Regarding oxidative stress and inflammation biomarkers in BALF and TA, we found concentrations of GSA with significantly higher values in the VAP group compared to the no VAP group (Table 5); however, no significant correlation was observed between values in BALF and TA (Pearson’s coefficient 0.12, *p* value 0.7).

**Table 5:** Median (P10–P90) metabolite concentrations in bronchoalveolar lavage fluid (BALF) and tracheal aspirate (TA) in patients with and without ventilator-associated pneumonia (VAP).

	BALF			TA		
Biomarker (nmol/L)	VAP (*N* = 7)	No VAP (*N* = 11)	*P*	VAP (*N* = 10)	No VAP (*N* = 7)	*P*
GSH	49 (16–488)	89 (6–426)	0.9	1811 (765–28,514)	332 (46–7367)	0.09
GSSG	27 (3–142)	41 (n.d. –187)	0.7	1927 (264–8435)	406 (63–2747	0.2
GSA	20 (n.d. –284)	n.d. (n.d.–n.d.)	0.04	3712 (n.d. –8683)	n.d. (n.d. –705)	0.03
Cystine	9 (n.d. –430)	2 (n.d. –155)	0.7	103 (n.d. –5047)	31 (n.d. –2977)	0.6
Methionine	201 (27–1663)	36 (n.d. –367)	0.05	23310 (425–53,349)	5711 (n.d. –18,305)	0.4
SAM	n.d. (n.d. –83)	n.d. (n.d. –32)	0.3	354 (n.d. –817)	n.d. (n.d. –1471)	0.7
3Cl-Tyr	n.d. (n.d. –1.4)	n.d. (n.d. –n.d.)	0.8	34 (n.d. –383)	n.d. (n.d. –37)	0.2
3NO_2_-Tyr	n.d. (n.d. –2)	n.d. (n.d. –0.7)	0.6	n.d. (n.d. –135)	10 (n.d. –43)	0.5

*n.d.* not detected.

*P* values correspond to Wilcoxon rank-sum test.

## Discussion

VAP constitutes one of the most serious complications of MV in preterm newborns. Nevertheless, the lack of a specific definition for neonates and the difficulties in obtaining uncontaminated samples of the lower respiratory airway in very preterm infants render microbiologic diagnosis and etiologic treatment extremely difficult.^12^ In this scenario, the availability of rapid and reliable biomarkers to diagnose VAP would be highly desirable.^13^ Millo et al., described a compartmentalized production of cytokines within the lung that is not apparent in the systemic circulation.^14^ According to these studies, biomarkers should be determined in respiratory samples, being BALF more suitable than TA for microbiologic diagnosis although it is technically more difficult to obtain. However, some authors have proposed the use of TA samples for the diagnosis of VAP.^15^ Notwithstanding, BALF is a highly reliable technique that may be more helpful in yielding potential VAP pathogens than tracheal aspirates.^5^ In this context, it is important to establish the difference between contamination and infection. Therefore, the confluence of clinical and analytical data that accompany microbiological data is necessary.^13^

Human pulmonary alveolar epithelial cells release interleukin-8 among other pro-inflammatory cytokines after stimulation by lipopolysaccharide.^16^ Studies in the adult population have evaluated the reliability of some of these pro-inflammatory cytokines (G-CSF, MIP-1α, IL-6, STREM, IL-8, and IL-1β) in BALF and identified TNF-α, IL-1 α, IL-1β, IL-8, and IL-6 as the most trustworthy biomarkers to diagnose VAP.^6,9^ Interestingly, IL-6 was found to discriminate VAP from other causes of pulmonary infiltrates.^17^ Likewise, IL-8 and TNF-α detection in BALF has been shown to be useful for monitoring the response to antibiotic treatment in adults.^10^ Nevertheless, the study of BALF cytokines in newborns has been limited within the frame of BPD,^18^ and hence, it cannot be ruled out that the rise of cytokines is caused exclusively by BPD or it is triggered by an infectious agent.^19^ On the other hand, currently there are no published studies characterizing BALF cytokine levels in preterm infants with VAP diagnosis.

Pro-inflammatory cytokines induced by innate immune response are mainly produced in activated lymphocytes and macrophages and are involved in regulating inflammation through cellular proliferation and differentiation, chemotaxis, and modulation of immunoglobulin secretion.^20^

In this scenario, TNF-α, a glycopeptide produced by macrophages, monocytes, and T-lymphocytes, is a central player in the pathogenesis of inflammation and autoimmune diseases and can trigger several inflammation-related cytokines and chemokines.^21^ This factor is essential in the development of the cytokine storm, which is present in bacterial and viral infections,^20,22^ including SARS-CoV-2.^23^ When infection occurs, TNF-α develops a pro-inflammatory action by itself and through the upregulation of other inflammatory mediators, such as IL-1 and IL-6.

TNF-α signaling occurs through two membrane receptors, i.e., TNFR1 and TNFR2.^24^ TNFR1 is expressed in nucleated cells, while TNFR2 is highly regulated and is only expressed in immune, endothelial, and nerve cells.^24–26^ TNFR1 contains a cell death domain that mediates apoptosis, while TNFR2 lacks this domain and cannot mediate this phenomenon. TNFR1 induces programmed cell death through the caspase-mediated apoptosis pathway.^24–26^ In addition, the binding between TNF-α and TNFR1 triggers intracellular signaling that ends with the activation of the transcription factor NF-κB. Once NF-κB is released from its inhibitors through multi-kinase-mediated phosphorylation, it is translocated to the cell nucleus, and expresses genes involved in protein synthesis related to the maturation of the innate immune response, adaptive response, inflammation, and autoimmunity.^27^

To the best of our knowledge, TNF-α in BALF has not been previously evaluated, neither in adult nor newborn populations as an early marker of VAP. However, there are some studies in adults in which TNF-α levels in the blood have been shown useful to diagnose VAP^28^ and in BALF could be useful to monitor inflammatory response.^29^ In addition, numerous cytokines have been investigated in TA^30–32^ and BALF^33,34^ for the diagnosis and evolution of pulmonary pathology such as BPD. The results of our study indicate that TNF-α could be an early predictive marker of lung infection and under prolonged MV, it could help neonatologists with therapeutic decisions. In addition, we found significant correlations between concentrations of IL-6 and TNF-α in BALF and TA in the second cohort. This could be a relevant finding that, if confirmed, would allow the use of non-invasive samples for the diagnosis of VAP in preterm infants.^35^

The study of oxidative stress biomarkers in the blood is a promising field in the diagnosis of infections in the neonatal stage.^36–38^ This study has managed to identify differences in biomarkers of inflammation (i.e., GSA) in BALF and TA samples, with similar results to those reported in other articles.^11^ Thus, it seems that GSA could be useful for the early diagnosis of VAP in newborns.

We understand that the reduced number of patients constitutes the main limitation to overtly ascribe TNF-α significance for being a reliable and early marker of VAP. Although no significant differences were observed in terms of days of MV and lung pathology between preterm infants with and without VAP, we cannot exclude that this cytokine response could also be associated with other types of lung injury, such as ARDS and/or MV. A much larger sample would be needed to sort this out, and to identify which cytokines might be associated with what types of lung injury. Besides, studies on the limit of detection and reproducibility of TNF-α in patients with confirmed bacteriologic VAP are warranted. On the other hand, one of the strengths of the study is the validation of the results in a second, independent cohort and employing a different analysis kit. Although some studies have found differences in cytokine concentrations in TA following administration of systemic corticosteroids in preterm infants, in our case, none of our patients had received systemic steroids prior to sample collection;^39^ hence, this factor does not affect our results.

## Conclusions

TNF-α in BALF and GSA in BALF and TA have shown to be associated with VAP in preterm newborns, thus they could be used as early biomarkers of VAP. Our data did not support the use of cytokines in TA instead of BALF for the diagnosis of VAP. However, given the limited number of patients studied, we warrant a prospective observational study including a sufficiently big number of VAP-diagnosed newborn infants.

## Supplementary information

Supplementary material

## Data Availability

All data generated or analyzed during this study are included in this article and/or its Supplementary material files. Further enquiries can be directed to the corresponding author.
