# Supplementary material for: Early molecular markers of ventilator-associated pneumonia in bronchoalveolar lavage in preterm infants
Source: Pediatr Res. 2022 Sep 7;93(6):1559–65. doi: 10.1038/s41390-022-02271-w (PMC9451119; doi:10.1038/s41390-022-02271-w)
Supplement: Supplementary file 1 — Supplementary material [file 41390_2022_2271_MOESM1_ESM.pdf]

Online Supplementary Material for

Early molecular markers of neonatal ventilator-associated pneumonia in bronchoalveolar lavage

Alejandro Pinilla-Gonzalez, MD[1,2]; Inmaculada Lara-Cantón, MD[1,2]; Laura Torrejón Rodríguez, MD[1,2]; Anna Parra-Llorca, MD, PhD[1,2]; Marta Aguar, MD, PhD[1,2]; Julia Kuligowski, PhD[2]; José David Piñeiro-Ramos, MsC[2]; Ángel Sánchez-Illana, PhD[2]; Ana Gimeno Navarro, MD[1,2]; Máximo Vento, MD,PhD[1,2,3]; María Cernada, MD,PhD[1,2]*

[1]Division of Neonatology. University and Polytechnic Hospital La Fe, Valencia, Spain.
[2]Neonatal Research Group, Health Research Institute La Fe, Valencia, Spain.
[3]National Coordinator of the Spanish Maternal and Infant Health and Development Network, Health Research Institute Carlos III, Spanish Ministry of Economy and Competitiveness (RD12/0026)

Analytical standards and reagents

Cystine, glutathione (GSH), glutathione disulfide (GSSG), methionine (Met), S-adenosylmethionine (SAM), 3-Cl-tyrosine (3Cl-Tyr), and 3-NO_2-tyrosine (3NO_2-Tyr), with purities ≥ 98% from Sigma Aldrich Química S.L. (Madrid, Spain) were employed as standards. Glutathione sulfonamide (GSA) was synthetized, purified and quality checked according to a previous work [1] and employed also as analytical standard. Phenylalanine-D_5 (Phe-D_5), and *p*-tyrosine (*p*-Tyr-D_2) were obtained from CDN Isotopes (Pointe-Claire, Canada) and Cambridge Isotope Laboratories (Tewksbury, MA, USA), respectively, and employed as internal standards (IS).

Perchloric acid, acetonitrile (LC-MS grade) (ACN), and N-Methylmaleimide (97 %) (NEM) were purchased from Sigma Aldrich Química SL (Madrid, Spain) and formic acid (98%) (FA) from Panreac (Barcelona, Spain). Water (H_2O) was Milli-Q grade provided by a Milli-Q Integral purification system from Sigma Aldrich Química SL (Madrid, Spain). Dulbecco's phosphate buffered saline (PBS) containing 137 mM of NaCl, 2.7 mM of KCl, 10 mM of sodium hydrogen phosphate, and 1.8 mM of potassium dihydrogen-phosphate at pH 7.4 was prepared by dissolving the pre-weighed tablet from VWR (Radnor, PA, USA) in 100 mL of H_2O.

Individual stock solutions from each standard were prepared by accurately dissolving the compounds in H_2O (0.1% FA) except for GSH which was dissolved in 100 mM NEM in PBS solution. Individual working solutions at 1 mM were prepared by dilution in H_2O (0.1% FA). For external calibration, standard solutions obtained from serial dilution in H_2O (0.1% FA) were used.

Sample processing

First, 50 µL of BALF and 200 µL of TA samples were thawed on ice and the aqueous internal standard (IS) mixture (i.e., Phe-D_5, *p*-Tyr-D_2) was added to reach a final concentration of 250 nM of each IS. Thereafter, samples were frozen at -80 °C in 2 mL microcentrifuge tubes and placed in the drying chamber of the miVac SpeedTrap from SP Scientific (Warminster, PA, USA) in a freeze drier configuration. Once sample lyophilization was completed (approximately after 8 hours), the obtained powder was dissolved in 90 µL of 10 nM NEM aqueous solution and incubated during 5 min. Afterwards, samples were deproteinized by the addition of 10 µL of aqueous perchloric acid (44 % w/v) followed of centrifugation at 10000 g at 4 °C during 15 min. Finally, the supernatants were injected into the UPLC-MS/MS system.

UPLC-MS/MS analysis

For the analytical determination of cystine, GSH, GSSG, GSA, Met, SAM, 3Cl-Tyr, and 3NO_2-Tyr, an Acquity-Xevo TQS system from Waters (Milford, MA, USA) was employed. Chromatographic separation conditions as well as mass spectrometric parameters were selected to achieve optimal separation and sensitivity during the injection of 1 µM individual standards. Separations were carried out employing an Acquity UPLC® BEH C_8 1.7 µm 2.1 x 100 mm column and an H_2O (0.1% FA):ACN (0.1% FA) binary gradient as follow: isocratic conditions during 1.25 min at 99 % of H_2O (0.1% FA); linear increase to 98 % of ACN (0.1% FA) during 3.5 min; maintaining 98% of ACN (0.1% FA) during 0.25 min; return to initial conditions in 0.1 min; maintaining 99 % of H_2O (0.1% FA) for system re-equilibration during 0.9 minutes. Multiple reaction monitoring (MRM) and positive electrospray ionization (ESI^+) were used. The ESI settings employed were source and desolvation temperatures at 150 ºC and 380 ºC, respectively; cone, desolvation, and collision gas flows at 150 L/hour, 800 L/hour, 0.15 mL/min, respectively; nebulizer pressure of 7 bars; dwell times were set to 5 ms to achieve a minimum of 10 points per peak. The quantification of the analytes was carried out by internal standard calibration of the integrated peaks areas employing a MassLynx 4.1 software from Waters (Milford, MA, USA). Calibration lines by linear regressions were calculated except for SAM and GSH, where degree-two polynomial fits were used and the limit of quantification (LOQ) was calculated as the lowest standard concentration used in the calibration line. The acquisition parameters and main figure of merit are shown in Table S1.

Table S1. Acquisition parameters and main figures of merit. Note: CE is Collision Energy, RT is Retention time, LOQ is Limit of Quantification, BALF in bronchoalveolar lavage fluid, TA is tracheal aspirate, and R^2 is the coefficient of determination.

**Metabolite**	**m/z Parent ion**	**Cone [V]**	**CE [eV]**	**m/z Daughter ion**	**RT ± s (min)**	**Calibration range (nM)**	**LOQ (nM)**		**R^2**	**Internal Standard**
							**BALF**	**TA**		
Cystine	241.2	20	15	120	0.509 ± 0.004	4 - 250	8	2	0.96	*p*-Tyr-D_2
GSH-NEM	433.1	25	20	201	2.350 ± 0.002	6 - 3000	12	3	0.997	Phe-D_5
GSA	338.0	45	25	155.1	0.608 ± 0.015	31 - 1000	62	16	0.994	*p*-Tyr-D_2
GSSG	613.2	50	35	355	0.754 ± 0.005	78 - 10000	156	39	0.992	*p*-Tyr-D_2
Met	150	30	25	60.7	0.711 ± 0.003	16 - 2000	31	8	0.990	*p*-Tyr-D_2
SAM	398.9	25	17	250.2	0.508 ± 0.005	4 - 1000	8	2	0.98	*p*-Tyr-D_2
3Cl-Tyr	216	30	15	170	1.378 ± 0.008	4 - 2000	8	2	0.998	Phe-D_5
$3NO_2$-Tyr	227.1	25	10	181	1.663 ± 0.006	2 - 1000	4	1.0	0.998	Phe-D_5
Phe-D_5	171.5	30	20	125	1.468 ± 0.008	—	—	—	—	—
p-Tyr-D_2	184.1	20	10	138.1	0.84 ± 0.02	—	—	—	—	—

References

1. Cháfer-Pericás C, Stefanovic V, Sánchez-Illana Á, Escobar J, Cernada M, Cubells E, et al. Novel biomarkers in amniotic fluid for early assessment of intraamniotic infection. Free Radic Biol Med. 2015;89:734-40.
